# Introduction of an Educational Video to Enhance the Informed Consent Process in Postoperative Radiation Therapy of Breast Cancer Patients

**DOI:** 10.3390/cancers16203552

**Published:** 2024-10-21

**Authors:** Samuel M. Vorbach, Martin Pointner, Jens Lehmann, Julian Mangesius, Tilmann Hart, Claudia Gstir, Theresa Rändler, Thomas Seppi, Ute Ganswindt, Siegfried Kollotzek

**Affiliations:** 1 Department of Radiation Oncology, Medical University of Innsbruck, 6020 Innsbruck, Austria; samuel.vorbach@i-med.ac.at (S.M.V.);; 2 Department of Psychiatry, Psychotherapy, Psychosomatics and Medical Psychology, University Hospital of Psychiatry II, Medical University of Innsbruck, 6020 Innsbruck, Austria

**Keywords:** informed consent, educational video, Delphi method, radiotherapy, breast cancer

## Abstract

Background/Objectives: Informed consent is crucial in medical practice, especially for complex treatments such as postoperative radiotherapy for patients with breast cancer. Conventional consent procedures are often based on verbal declarations with a highly condensed but nevertheless large amount of information, which can exceed the recording capacity of patients and lead to misunderstandings. The aim of this study was to develop and test an educational video on breast cancer patients to enhance the informed consent process by improving patients’ understanding and reducing the duration of the subsequent consultation. Methods: The educational video was created after the underlying content was determined by a modified Delphi method in which a panellist of radiation oncologists, nurses, radiation therapists, and former patients participated in successive rounds of topic scoring. After achieving content consent, the video included 19 items to cover key aspects of postoperative radiotherapy in a patient-friendly manner. Fifty breast cancer patients scheduled for postoperative radiotherapy were randomised to watch the video prior to their verbal consultation (*n* = 25) or to the control group participating in the traditional informed consent process only (*n* = 25). The duration of the informed consent processes in both arms was recorded. To assess the educational effect of the video, a multiple-choice test was created. In addition, patients’ satisfaction was recorded using a separate questionnaire. Both questionnaires were completed by the patients one to two hours after signing the consent form. Results: The patients in the video group evidenced significantly higher knowledge scores than those who received standard verbal explanations (median number of correct answers 9 vs. 8 out of 10, *p* = 0.0039). The whole informed consent process was also completed faster in the video group (mean duration 34.7 vs. 46.2 min, *p* < 0.001). Median satisfaction scores were high in both groups (34 vs. 33 out of 35 points), with no significant differences observed (*p* > 0.05). Conclusion: The educational video effectively improved patient understanding and streamlined the informed consent process without compromising patients’ satisfaction. This approach also helps to standardise the delivery of complex medical information, and it can also be adapted to improve the informed consent process for other cancer treatments.

## Simple Summary

Our research addresses the challenges in informed consent in postoperative radiotherapy for breast cancer. Traditional consent processes often rely on verbal explanations of sometimes complicated treatment options, which can overwhelm patients and lead to misunderstandings. To improve this fundamental process, we developed an educational video using a modified Delphi method, incorporating input from experts and former patients to ensure comprehensive and patient-friendly content. With our study, we demonstrated that this video, if viewed by breast cancer patients prior to their subsequent verbal consultation, can significantly improve patients’ understanding and speed up the informed consent process without compromising patients’ satisfaction. To conclude, this innovation supports a more comprehensive and efficient informed consent process, which is in line with evolving healthcare practices that prioritise patient engagement and education.

## 1. Introduction

Informed consent is a fundamental ethical and legal requirement in medical practice [1,2]. A health intervention can only be performed after the patient has given free and informed consent [3]. The condition for valid consent is a transparent and thorough information process [4] that includes the disclosure and understanding of information [5]. Informed consent is particularly crucial in cancer treatment, where patients face complex decisions with significant implications for their health and quality of life [6,7]. In breast cancer treatment, postoperative radiotherapy plays a vital role by reducing the 10-year local recurrence risk by about 50% and the 15-year breast cancer death rate by about one-sixth after breast-conserving surgery [8,9,10]. However, the informed consent process for this treatment can be challenging due to the technical nature of radiotherapy, potential side effects, and the emotional stress patients experience following breast cancer diagnosis and surgery [11,12]. Traditional approaches to informed consent often rely heavily on verbal explanations during medical consultations, which may lead to information overload, misunderstandings, or incomplete retention of important details [13,14].

To address all these challenges, there has been growing interest in developing multimedia educational tools to supplement and enhance the informed consent process [15,16]. Educational videos, in particular, have shown promise in improving patient knowledge [17], reducing anxiety regarding their upcoming treatment [18], and increasing satisfaction [19,20] with the consent process across tens of medical and surgical procedures [17,21,22]. As reported, for example, educational videos were demonstrated to effectively help trauma patients and their family members obtain adequate knowledge for making a rational treatment decision even under stressful conditions [19]. This is an important finding, since patients should feel themselves encouraged and also actively be engaged in their health decision-making process [23]. Finally, a more effective informative process might provide more resources for the clinicians to explore the patient’s perspective in terms of apprehension, interrogations, and barriers to treatment, in order to potentially enhance treatment adherence and success of care [24].

Among neoplastic diseases in women, breast cancer has the highest incidences [25], and it continues to rise [26]. Accordingly, postoperative radiotherapy for this large and growing cohort of patients also requires measures to improve routine clinical practice, of which the educational discussion is the first crucial part. Our aims were therefore to (1) create a comprehensive educational video to inform breast cancer patients in advance about postoperative radiotherapy, and to (2) test whether the use of the supplementary video at the beginning of their first radiation oncology consultation can increase patient understanding and satisfaction and possibly even shorten the duration of the educational process.

## 2. Materials and Methods

### 2.1. Development of the Educational Video

The initial part of the study comprised the development of an educational video for breast cancer patients undergoing postoperative radiation therapy. The goal was to tailor its content by including comprehensible and relevant information about the treatment of this specific type of malignancy, its unique procedures, alternative options, and its benefits and risks. The development of the educational video was a multi-step process:

1. To develop the video content, we assembled a panel of experts, including eight radiation oncologists, three radiology technologists, two nurses, and three voluntary breast cancer survivors who had undergone postoperative radiation therapy in the last 2 years (see Table 1). Recruitment of health care professionals was performed within our department on the basis of voluntary participation. Breast cancer patients who had recently been treated in our department were invited by email to join the expert panel.
2. We used a modified Delphi technique to reach accelerated consensus among the members of our expert panel by reducing the number of feedback rounds from typically four or more to three rounds. In addition, the full anonymity of the participating experts—another characteristic of a traditional Delphi method—was not mandatory. The process started by drafting a script that covered the aforementioned key aspects of breast cancer radiation therapy. For this purpose, the experts’ individual draft contributions were collected through written submission and compiled by the facilitator thereafter. The experts were then asked to review the first draft online and indicate which elements they thought were indispensable for patients to be comprehensively informed about in their upcoming treatment. In a second online round, the experts ranked the pertinence of each item by using a 5-point Likert scale (1 to 5, with 5 indicating the highest pertinence). Consensus for item inclusion was defined as reaching a median item score above 3.0. The experts then had the opportunity to suggest new items in a free-text section. A similar ranking was carried out online in the final round, where a median score of > 3.5 was defined as sufficient consensus. Rating contributions of patients and healthcare professionals were weighted equally.
3. Video production: Given patient preferences and the need to avoid information overload, the experts agreed that the duration of the video should be approximately 10 min. Once the script was finalized, the video was produced in collaboration with our in-house multimedia company. The focus was on patient-friendly language, and visual aids were used to explain complex medical procedures and concepts.

### 2.2. Multiple-Choice Test

The second component of our study was the development of a knowledge assessment tool. Our literature review revealed that there is no instrument specifically designed to quantify breast cancer patients’ knowledge of postoperative radiotherapy as a prerequisite for informed consent. For this purpose, the Delphi expert panel also developed a suitable knowledge assessment tool. It included ten multiple-choice questions on primary aspects of postoperative radiotherapy, with each question being weighted equally. 

Initially, the individual experts contributed questions on their own and then discussed them collectively in the expert panel. We agreed on a selection of 12 multiple-choice questions. We tested the question pool on a sample of ten breast cancer patients currently undergoing postoperative radiotherapy who had already consented to radiotherapy after discussing it with their doctor. Wrongly answered questions that turned out to be too specific or that required advanced expert knowledge to be answered were eliminated from the pool. This was the case for two questions, each with 70% incorrect answers. In addition, we also tested our questions on ten patients who had yet to have their informed consent discussion. Questions that were answered correctly by all (100% of these patients) were designated to be excluded from the pool. However, none of the questions met this exclusion criterion. In total, ten questions were included in our knowledge assessment tool (see Supplementary S1).

### 2.3. Patient Satisfaction Assessment

Patient satisfaction with the information process was assessed using a 7-point questionnaire. To our knowledge, a questionnaire specifically addressing satisfaction with the informed consent process is not available. The available PATSAT-C33 instrument to measure patient satisfaction could not be completely used in our study, since it addresses overall patient satisfaction regarding their treatment and/or clinical stay. Thus, only three questions out of 33 were take from the validated PATSAT-C33 questionnaire [27]. These three questions addressed satisfaction with the obtained information on the patient’s disease and treatment as well as the time devoted to the patient. In addition, our questionnaire included four questions that have been self-developed by the expert panel. These questions aimed to assess the opportunity to ask questions, the overall satisfaction with the consent process, comprehensibility of information, and support in the patient’s decision-making. Satisfaction with various aspects was rated on a Likert scale ranging from 1 to 5 points (see Supplementary S2). 

### 2.4. Study Designed to Test the Educational Video in Clinical Care

We aimed to recruit 50 patients with breast cancer scheduled for postoperative radiation therapy (RT) at the Department of Radiation Oncology of the Medical University of Innsbruck. The power analysis is based on the following assumptions: (a) the video reduces the mean time for the informed consent process (including anamnesis, physical examination, photographic documentation of the breast, and the informed consent discussion) by 10 min (37.5 min to 27.5 min); (b) the standard deviation is 10 min for each group; and (c) the level of significance is 0.05 (*p* < 0.05). Given these assumptions, a sample size of at least 34 was required to achieve 80% power with a significance of 0.05. Due to the greater variability in the duration of the initial consultation, as determined by clinical experience in the past, the target sample size to be analysed was increased to 50 patients. 

Patients were approached consecutively and considered eligible if they (1) received postoperative RT for breast cancer, and (2) had sufficient German language skills to understand the video and to complete the questionnaires. We assessed patient demographic data including age, sex, education, professional status, computer, and internet skills. To avoid bias, patients were excluded if they were or had a family member who was a healthcare professional. Patients with assessed cognitive impairment were also excluded.

Patients that met the inclusion criteria were informed about the study during their first appointment and, if they wished to participate, were randomly allocated by dicing to either the video group or the control group participating in the traditional informed consent process only. After being presented with the video (video group) in a designated room in our department and completing the informed consent process (both groups), all participants were asked the following:

1. To complete the multiple-choice test to assess their understanding of crucial aspects of postoperative radiotherapy for breast cancer;
2. To complete the patient satisfaction assessment regarding the informed consent process.

Both questionnaires were completed in privacy by the participants in a designated room in our department one to two hours after signing their consent form.

### 2.5. Ethics

The study was approved by the institutional review board of the Medical University of Innsbruck (EC No. approval: 1343/2023). All procedures conducted in this study involving human participants were in accordance with the ethical standards of the institutional review board as well as with the Helsinki Declaration (1964) and its later amendments or equivalent ethical standards. 

### 2.6. Statistical Analysis

Descriptive statistics were utilized to depict the basic characteristics of study participants and experts. Video items rated by the expert, patient satisfaction scores, and time required for the informed consent process are reported as mean or median and range values. The Mann–Whitney U test was used to compare participants’ satisfaction (regarding the informed consent process), and the multiple-choice test results of the video group versus the control group. An independent *t*-test was performed to assess the differences in the duration of the informed consent process (in the video group versus the control group). For intra-cohort comparison of age, distribution of tumour site occupational status, degree of education, and usage of electronic media devices, appropriate statistical tests were applied (Mann–Whitney U, Chi-square test, or Fisher’s exact tests). Statistical analysis was conducted using SPSS Statistics (V26, IBM Cooperation, Armonk, NY, USA). The *p* values < 0.05 were considered to be significant. 

## 3. Results

### 3.1. Composition and Characteristics of the Delphi Expert Group

The expert panel consisted of 16 participants, including 8 physicians, 3 radiology technologists, 2 nurses, and 3 patients who had undergone radiation therapy. The experts had different levels of work experience, ranging from less than 10 years to more than 40 years. Age distribution was well-balanced. Ten out of sixteen experts were women.

### 3.2. Delphi Process

The modified Delphi process consisted of three rounds. In the beginning, each of the panellists defined a first set of topics for the pool of items to be included in the video. In total, 20 different items were identified. 

Four topic items (No. 1.0, 2.2, 2.5, and 6.0) did not surpass the necessary median Likert scale scoring of > 3.0 and were eliminated from the pool after the first evaluation round. By contrast, five new items (2.6, 2.7, 2.8, 3.4, and 8.0) were added, all of which achieved high consensus in the final round of evaluation, with a median item score > 4.0.

For this last evaluation round, the consensus threshold was raised to a median item score of > 3.5. Two items (2.4 and 3.3) did not reach the consensus threshold in the final round and were eliminated. In total, 19 items reached the consensus threshold in the final round. These items cover a wide range of aspects, including treatment procedures, acute and late side effects, patient care, and follow-up, providing a comprehensive framework for the educational video content. Evaluation results are summarized in Table 2.

### 3.3. Characteristics of the Patients in the Pilot Study

Between February and April 2024, we approached 54 consecutive breast cancer patients who met the inclusion criteria, and 50 patients (92.6%) agreed to participate in the study, of whom 25 patients were randomly assigned to the educational video group and 25 to the control group with conventional education. The characteristics of the study participants are shown in Table 3.

The median age of all participants was 60 years (range: 40–81). The median age in the control group was 55 years (range: 40–81), while the median age in the video group was 62 years (range: 43–72). Median age did not differ significantly between the two groups. (Mann–Whitney, U = 245.5, *p* = 0.193). 

The distribution of tumour sites was balanced within the overall sample (*n* = 50), with 50% of tumours located in the left breast and 50% in the right breast. However, the distribution was significantly different between the two study cohorts (χ^2^ (df = 1, *n* = 50) = 3.92, *p* = 0.048). In the control group video, 36% of patients had left-sided tumours and 64% had right-sided tumours, whereas for patients in the video group it was exactly the opposite (64% left-sided and 36% right-sided tumours). Irradiation of peripheral lymph nodes was indicated equally often in the video group and in the control group (7 patients in each group, 28%). 

In total, 48% of all patients were employed and 52% were retired (control group: 60% versus 40%; video group: 36% versus 64%). The difference in occupational status between the two study groups was not statistically significant, as determined by a Chi-squared test, χ^2^(df = 1, *n* = 50) = 2.885, *p* = 0.089. 

In the overall sample, 20% of patients had compulsory education (control group: 20%, video group: 20%), 46% received vocational training (44% versus 48%), 12% disposed of a high school degree (8% versus 4%), and 22% had a university degree (28% versus 16%). Fisher’s exact test was used to examine the association between study group patients and educational attainment. The result was not statistically significant (*p* = 0.380). 

Most participants owned various forms of electronic media equipment. Smartphone ownership was almost universal (96%). In addition, 48% of participants reported owning a laptop or computer, 30% had access to a tablet, and 88% used email. Of the latter, 54.5% used email daily, 20.5% used it once a week, 6.8% used it every two weeks, and 18.2% used it less frequently. Availability and usage frequency of electronic media and communication equipment were not significantly different between the study groups (*p* values ranging from 0.185 to 0.758). 

### 3.4. Results of the Pilot Study

#### 3.4.1. Multiple-Choice Test

Patients in the video group (median number of correct answers: 9, range 6–10) achieved a significantly higher score than patients in the control group (median number of correct answers: 8, range 3–10) (U = 212.5, *p* = 0.039). The distribution of correct and incorrect answers per question between the two study groups is shown in Table 4.

No difference in the multiple-choice test scores depending on the level of patients’ education could be detected within the entire cohort (pooled group A—compulsory or vocational training; median score: 8 [range 3–10], pooled group B—high school or university degree; median score: 8 [range 6–10]; Mann–Whitney, U = 258.5, *p* = 0.632). Exploratory analyses of multiple-choice test results within the video and the control group revealed no statistically significant difference depending on the degree of education (video subgroup A: median score 8 [range 3–10], video subgroup B: median score 8 [range 6–9]; Mann–Whitney, U = 67.0, *p* = 0.951). A corresponding result was observed for the control group (control subgroup A: median score 9 [range 6–10], control subgroup B: median 8.5 [range 7–10]; Mann–Whitney, U = 64.0, *p* = 0.623).

#### 3.4.2. Patient Satisfaction Assessment

**Figure 1:**
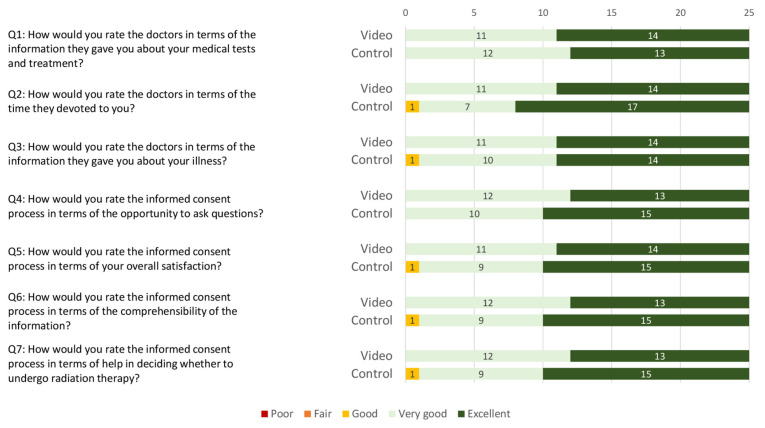
Stacked bar chart of patient satisfaction scoring regarding the informed consent process in the video and the control group. Ratings are categorised on a 5-point Likert scale (i.e., poor, fair, good, very good, and excellent).

Overall satisfaction with the informed consent process was high in both groups. The median overall satisfaction score was 34 (range: 28–35) in the video group and 33 (range: 23–35) in the control group (Figure 1). No statistically significant difference in patient satisfaction was found between the two groups for the three questions taken from the PATSAT-C33 questionnaire (*p* = 0.950) and the four self-developed questions (*p* = 0.863).

#### 3.4.3. Duration of the Informed Consent Process

**Figure 2:**
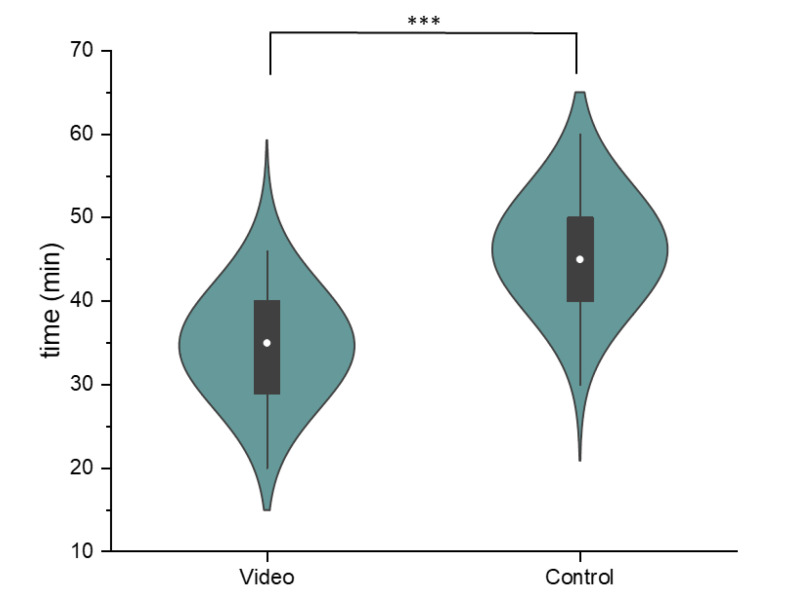
Comparison of the duration of the informed consent process for the video and the control group. The violin plots show the normally distributed data, while the box plot indicates the median (white dot), the interquartile range (black box), and the range of data (whiskers) (*** indicates the *p*-value < 0.001 in the independent *t*-test).

The mean duration of the informed consent process in the video group was 34.7 min (SD = 7.6, Figure 2) and 46.2 min in the control group (SD = 7.8). An independent *t*-test showed statistically significant difference between the two groups, *t*(48) = 5.304, *p* < 0.001. 

The analysis of differences in the duration of the informed consent process depending on the level of education revealed no significant deviations within the entire patient cohort (pooled group A—compulsory or vocational training; mean duration: 40.4 min [SD 10.2 min], pooled group B—high school or university degree; mean duration: 40.4 min [SD 8.4 min]; *t*(48) = 0.004, *p* = 0.997). The separate analyses of the duration assessments within the video and the control group revealed no statistically significant difference depending on the degree of education (mean duration of video subgroup A: 34.1 min [SD 7.3 min], mean duration of video subgroup B: 46.0 min [SD 8.4 min]; *t*(23) = −0.592, *p* = 0.560). An analogous result was observed for the control group (control-subgroup A mean duration: 47.2 min [SD 8.4 min], control-subgroup B mean duration: 44.3 min [SD 8.5 min]; *t*(23) = 0.879, *p* = 0.388).

## 4. Discussion

Our pilot study demonstrated that patients who viewed the educational video before their consultation scored significantly higher on knowledge than those who received standard verbal explanations (median number of correct answers 9 out of 10 vs. 8 out of 10; see Table 4). Our video was successful in adequately informing patients about postoperative radiotherapy, and thus they might be better prepared to engage more effectively in the subsequent decision-making discussion. 

As a whole, the design of the informed consent process was well received by all patients, regardless of whether they were assigned to the video or control group, which is reflected in equally high satisfaction scores achieved in both groups (see Figure 1). Moreover, the duration of the entire informed consent process (including medical history, physical examination, and photographic documentation of the breast) could be shortened by a mean of 11 min in the video group (see Figure 2). A more efficient informed consent process allows physicians to dedicate more time to patient care, which is increasingly important given restricted human resources [28] and rising cancer incidences [25,29]. Most importantly, pre-education of patients provides the clinician with the opportunity to more intensively explore the patient’s perspective in terms of fears, questions, and barriers to treatment. Finally, our combined findings suggest that pre-consultation education can streamline the process without compromising patient understanding. 

The complexity and challenges of informed consent have repeatedly been discussed over the last four decades [30,31]. Apart from the fact that patients show considerable variation in their interest and ability to absorb and understand information about their forthcoming treatment options, health care professionals are faced with the Herculean task of conveying every facet of their knowledge and expertise in the space of a 30-min consultation. As a result, an ideally informed consent process is difficult to define and, by its very nature, can only be aspired to rather than fully realised in everyday clinical practice.

A notable advantage of using educational videos is their ability to standardise parts of the informative process for patients, ensuring consistency across different healthcare providers. This approach not only enhances patient understanding but also reduces variability in the informed consent process, contributing to higher-quality care and legal compliance. Furthermore, videos can be produced in different languages or with added subtitles, which alleviates the transportation of crucial knowledge regarding their therapy, particularly to patients who do not dispose of adequate skills in the official language of their country. The implementation of audiovisual tools could help to ensure that such patients experience the same level of understanding and engagement in their treatment process as fellow native speakers. In conclusion, a harmonized informed consent process has the potential to decrease inequalities in healthcare. In principle, an educational video produced in a single centre and by nature tailored to its specific routines could be used in other clinics. Apart from generally valid content topics, it would probably need to be adapted to meet the different needs and available techniques of each external institution. For the international applicability of core content, the video must of course be translated to the local languages. In our breast cancer cohort, most patients owned various forms of electronic media equipment, such as smartphones, laptops, and tablets. These findings are in excellent agreement with those of Bach et al. [32], who evaluated the availability of internet resources as a prerequisite of e-Health applications for cancer populations. They assessed that in their cohort of 150 patients with gynaecologic malignancies, 94% had an internet access device. This nowadays widespread ownership suggests that the majority of cancer patients could easily access educational information provided by healthcare institutions already in the run-up of the informed consent discussion. Such measures can also prevent patients from using and being confused by inappropriate or less trustworthy sources in the wealth of health information available online, especially as these do not usually reflect the case-specific situation of an individual patient [33]. However, to ensure that actually all patients profit from essential educational material, it also has to be provided as an integral part of the in-site care process. In addition to any online availability, we thus recommend giving patients the opportunity to watch an educational video as the introduction to the informed consent discussion.

We did not assess patients’ baseline medical knowledge prior to the study inclusion, which could have influenced their test results as well as their satisfaction with the informed consent process with or without watching the educational video. In addition, we are aware that the patients’ understanding can never be fully assessed by a simple multiple-choice test with a restricted number of selected questions. Furthermore, it cannot be ruled out that the expert group had the video items in mind when creating the multiple-choice test. To minimise bias, it may therefore be advantageous to have the multiple-choice test developed by a separate group of experts from those who developed the items for the video. However, as a supporting source of information, the video should in any case contain only the identical content in condensed form that is also made available to patients in the classic information session without video support. In addition, it is difficult to recruit enough staff with the necessary expertise to form two different expert groups within a single clinic. With regard to the design of the informed consent process, patient satisfaction scores were equally high in both groups. However, this might be caused by an inherent bias, namely the reported tendency of patients to rate their healthcare providers very highly in general, especially if they are asked by their assigned physician to compile a satisfaction questionnaire—even if the questionnaire was submitted anonymously [34,35,36]. 

## 5. Conclusions

In conclusion, this study highlights the potential of video-based education to improve informed consent processes at the beginning of complex medical treatments like postoperative radiotherapy for breast cancer. By increasing patients’ understanding, these tools help to better meet ethical and legal requirements and to improve shared decision-making. In addition, the patients in our study responded very favourably to the introduction of the educational video.

Future research should investigate whether the provision of informative videos on education online platforms of official health care institutions can further improve the efficiency of informed consent processes in various medical disciplines.

## Figures and Tables

**Table 1:** Characteristics of the experts.

Characteristic		Value
Total no. of experts		16
Specialty	Physician	8
	Radiology technologist	3
	Nurse	2
	Former patient	3
Years of work experience (HCPs)	≤10	5
	11–20	2
	21–30	4
	31–40	1
	>40	1
Sex	Male	6
	Female	10
Age	20–29	1
	30–39	5
	40–49	6
	50–59	3
	>60	1
HCPs: Healthcare professionals		

**Table 2:** Delphi results.

Item No.	Topic	Rating Delphi Rounds 1/2 if Not (Re-)Evaluated Median
1.0	Biological effects of radiation on cancer cells and normal tissue	3/-
2.0	Radiation therapy: general procedure and workflow	5/5
2.1	Procedure: planning scan, skin marking	5/5
2.2	Cost of radiation therapy	1/-
2.3	Immobilisation device and couch	4/4
2.4	Verification imaging and couch adjustments	3.5/3.5
2.5	Linear accelerator: functionality, structure, safety	2.5/-
2.6	Number of radiation treatment sessions, sessions per week, appointment planning and requests	-/4
2.7	Duration of treatment session	-/4
2.8	Risk to small children or pregnant women in the vicinity after irradiation	-/5
3.0	Acute side effects: tiredness, fatigue	5/5
3.1	Acute side effects: skin irritation, itching, redness, dryness, open lesions	5/5
3.2	Acute side effects: breast/chest area pain, swelling	5/5
3.3	Acute side effects: stomach pain, loss of appetite	4/3
3.4	Skin care management during and after radiation therapy	-/5
4.0	Late side effects: skin darkening, poor wound healing, thin and dry skin, telangiectasia	5/5
4.1	Late side effects: lung pneumonitis, fibrosis	5/5
4.2	Late side effects: cardiac arrhythmias, cardiomyopathy, pericarditis, valvular dysfunction, coronary artery disease	5/4.5
4.3	Late side effects: breast hardening, feeling of tightness, reduction in breast size, pectoralis muscle shortening	5/5
4.4	Late side effects: lymphedema	5/4.5
4.5	Risk of secondary malignancy after radiation therapy	3,5/4
5.0	Reference to psycho-oncological care and art therapy	4/4
6.0	Virtual tour of the radiation oncology outpatient clinic	2/-
7.0	Oncological follow-up	4/4
8.0	Advice to contact the staff at any time in case of need/questions/symptoms	-/5

**Table 3:** Patient characteristics.

Characteristics	Overall	Without Video	With Video
Patients	50	25	25
Median age (range)	60 (40–81)	55 (40–81)	62 (43–72)
**Tumour site**			
Left	25/50	9/25	16/25
Right	25/50	16/25	9/25
**Radiotherapy to regional lymph nodes**			
Yes	14/50	7/25	7/25
No	39/50	18/25	18/25
**Occupational status**			
Employed	24/50	15/25	9/25
Retired	26/50	10/25	16/25
**Degree of education**			
Compulsory education	10/50	5/25	5/25
Vocational training	23/50	11/25	12/25
High school	6/50	2/25	4/25
University	11/50	7/25	4/25
Technical Equipment *			
Smartphone	48/50	23/25	25/25
Tablet	15/50	7/25	8/25
Laptop/Computer	24/50	11/25	13/25
Email	44/50	22/25	22/25
**Email use**			
Daily	24/44	11/22	13/22
Once per week	9/44	5/22	4/22
Every two weeks	3/44	1/22	2/22
Less frequently than every two weeks	8/44	5/22	3/22

* Multiple answers possible.

**Table 4:** Multiple-choice test results.

Question	Correct Answers/Incorrect Answers	
	Video	Control
Q1: Follow-up	23/2	14/11
Q2: Side effects	24/1	24/1
Q3: Duration of irradiation	18/7	16/9
Q4: Radiation treatment schedule	24/1	24/1
Q5: Target volume definition	15/10	16/9
Q6: LINAC	16/9	13/12
Q7: Skin markings	24/1	23/2
Q8: Preparation	24/1	22/3
Q9: Physical activity	24/1	22/3
Q10: Danger to others	25/0	23/2

## Data Availability

The data presented in this study are available on request from the corresponding author.

## Supplementary Materials

The following supporting information can be downloaded at: https://www.mdpi.com/article/10.3390/cancers16203552/s1.
