# Supplementary material for: Introduction of an Educational Video to Enhance the Informed Consent Process in Postoperative Radiation Therapy of Breast Cancer Patients
Source: Cancers (Basel). 2024 Oct 21;16(20):3552. doi: 10.3390/cancers16203552 (PMC11505877; doi:10.3390/cancers16203552)
Supplement: Supplementary file 1 [file cancers-16-03552-s001.zip › cancers-3251362-supplementary.pdf]

cancers MDPI

Supplementary S1 Patient satisfaction assessment

The original questionnaire was in German and was translated into English.

Experiences with the informed consent process

We are interested in your experience with the information session on radiotherapy for breast cancer. Please answer all the questions yourself and select the number that best applies to you. There are no 'right' or 'wrong' answers. Your information will be treated in strict confidence.

**In this hospital, how would you rate doctors in terms of:**					
	Poor	Fair	Good	Very good	Excellent
1. The information they gave about your medical tests and treatment?	1☐	2☐	3☐	4☐	5☐
2. The time they devoted you?	1☐	2☐	3☐	4☐	5☐
3. The information they gave you about your illness?	1☐	2☐	3☐	4☐	5☐
**How would you rate the information process in this clinic in terms of:**					
	Poor	Fair	Good	Very good	Excellent
4. The opportunity to ask questions	1☐	2☐	3☐	4☐	5☐
5. Your overall satisfaction with the informed consent process?	1☐	2☐	3☐	4☐	5☐
6. The comprehensibility of the information?	1☐	2☐	3☐	4☐	5☐
7. Help in deciding whether to undergo radiation therapy?	1☐	2☐	3☐	4☐	5☐

Supplementary S2 MC-Test

The original questionnaire was in German and was translated into English.

Pleas choose one Answer per Question.

1. Where do you receive your breast cancer follow-up care after radiotherapy?

a) at the clinic where you had your surgery

b) at the University hospital for Radiation Oncology

c) via your family doctor

d) no follow-up care is planned after the therapy

e) with any doctor

2) Which statement about the side effects of post-operative radiotherapy is true?

a) They are usually severe and permanent

b) They occur in every patient

c) They can include fatigue, skin irritation and swelling

d) They are usually noticeable immediately after radiotherapy

e) They can occur all over the body

3) Approximately how long will you be irradiated for each session?

a) 1-3 minutes

b) About 15 minutes

c) About one hour

d) More than two hours

e) The whole day

4) How often does a radiotherapy session typically take place?

a) Once a week (Mon)

b) Three times a week (Mon, Wed, Fri)

c) Five times a week (Mon-Fri)

d) Every other day

e) Daily (Mon-Sun)

5) How is the target volume for radiotherapy of the breast determined?

a) By the clinical experience of the radiation oncologist

b) By the position of the breast

c) By imaging before the start of treatment

d) The size of the breast

e) By the type of cancer

6) How is radiotherapy usually carried out?

a) Through a radiation source in the table on which you are lying

b) Via a radioactive mat which is placed on the chest.

c) Through a linear accelerator, from outside of the body (external irradiation)

d) Through a probe that is inserted into the breast

e) By an irradiation device held in the doctor's hand.

7. The markings on the skin after the planning CT should

a) Enable the patient to be positioned correctly for the radiotherapy

b) Make possible side effects visible

c) Simplify the identification of patients

d) Remind patients of the radiotherapy treatment

e) Be removed as quickly as possible

8) What should a patient consider before starting radiotherapy?

a) She should completely change her diet

b) She should avoid any physical activity

c) She should care for and protect her skin in the irradiated area

d) She should discontinue her long-term medication

e) She should have lymphatic drainages every day

9) How can a patient maintain her physical fitness during radiotherapy?

a) She should avoid intense exercise

b) She should continue with light to moderate exercise such as walking or yoga, as long as she feels well

c) She should start intensive strength training

d) She should stop all physical activity

e) She should exercise to her physical limit several times a week

10. After radiotherapy you

a) must not play with children for a few hours, as they could be endangered by you emitting radiation

b) may play with your children and grandchildren without concern

c) can endanger pregnant women through too close contact

d) must not use public transport

e) must not work
